# Developing Multimodal IoMT Monitoring System of Geriatric Depression: A Feasibility Study

**DOI:** 10.1093/geroni/igab046.2076

**Published:** 2021-12-17

**Authors:** Heejung Kim, Youngshin Cho, Kyuhee Lim, Sunghee Lee, Yuntae Kim, Mijung Kim

**Affiliations:** 1 Yonsei University, Seoul, Seoul-t'ukpyolsi, Republic of Korea; 2 BRFrame Inc., Seoul, Seoul-t'ukpyolsi, Republic of Korea; 3 Mapo Senior Welfare Center, Seoul, Seoul-t'ukpyolsi, Republic of Korea

## Abstract

The Internet of Medical Things (IoMT) is a promising tool to monitor depression and relevant symptoms. However, the multimodal IoMT monitoring system has been rarely developed considering the characteristics of older adults, particularly living in the community. Therefore, it is necessary to know how to develop multimodal IoMT monitoring systems tailored for older adults and evaluate the feasibility for research and practice. We developed a multimodal IoMT monitoring system that included a smartphone for facial and verbal expressions, smartwatch for activity and heart rates, and ecological momentary assessment (EMA) application. A convenience sample of 21 older Korean adults aged over 65 years was recruited from a community center, and 19 participants completed it. The data were collected in four weeks using self-report questionnaires, IoMT devices, and semi-structured interviews between July and December 2020 and were analyzed in mixed methods. Based on the Geriatric Depression Scale-Short Form scores, eight participants were classified in the depressive group (38.1%) and 13 in the non-depressive group (61.9%). A total of 1,505 (70.72%) EMA data were collected, and 1,277 (60.00%) were analyzed. Furthermore, 1,421 (66.78%) facial expression data were collected and labeled, including anger, happiness, neutral, sadness, surprise, and exception. Voice dialogues were transformed into 5,264 scripts. The depressive group showed lower user acceptance relative to the non-depressive group. However, both groups experienced positive emotions, had regular life patterns, and increased their self-interest. Thus, our multimodal IoMT monitoring system is a feasible and useful measure for acquiring mental health information in older adults’ depression.

